# A new benchmark of soft X-ray transition energies of $\mathrm {Ne}$, $\mathrm {CO}_2$, and $\mathrm {SF}_6$: paving a pathway towards ppm accuracy

**DOI:** 10.1140/epjd/s10053-022-00355-0

**Published:** 2022-03-01

**Authors:** J. Stierhof, S. Kühn, M. Winter, P. Micke, R. Steinbrügge, C. Shah, N. Hell, M. Bissinger, M. Hirsch, R. Ballhausen, M. Lang, C. Gräfe, S. Wipf, R. Cumbee, G. L. Betancourt-Martinez, S. Park, J. Niskanen, M. Chung, F. S. Porter, T. Stöhlker, T. Pfeifer, G. V. Brown, S. Bernitt, P. Hansmann, J. Wilms, J. R. Crespo López-Urrutia, M. A. Leutenegger

**Affiliations:** 1 Dr. Karl Remeis-Observatory and Erlangen Centre for Astroparticle Physics, Friedrich-Alexander-Universität Erlangen-Nürnberg, Sternwartstr. 7, 96049 Bamberg, Germany; 2 Max-Planck-Institut für Kernphysik, Saupfercheckweg 1, 69117 Heidelberg, Germany; 3 Institute of Theoretical Physics, Friedrich-Alexander-Universität Erlangen-Nürnberg, Staudtstr. 7/B2, 91058 Erlangen, Germany; 4 CNRS, Institut NEEL, Université Grenoble Alpes, CNRS, Institut NEEL, 25 rue des Martyrs BP 166, 38042 Grenoble Cedex 9, France; 5 CERN, 1211 Geneva 23, Switzerland; 6 Deutsches Elektronen-Synchrotron DESY, Notkestr. 85, 22607 Hamburg, Germany; 7 NASA Goddard Space Flight Center, 8800 Greenbelt Rd., Greenbelt, MD 20771 USA; 8 Lawrence Livermore National Laboratory, 7000 East Ave., Livermore, CA 94550 USA; 9 Institut für Optik und Quantenelektronik, Friedrich-Schiller-Universität Jena, Max-Wien-Platz 1, 07743 Jena, Germany; 10 Department of Astronomy, University of Maryland, College Park, MD 20742 USA; 11 Institut de Recherche en Astrophysique et Planétologie, 9, avenue du Colonel Roche BP 44346, 31028 Toulouse Cedex 4, France; 12 Ulsan National Institute of Science and Technology, 50 UNIST-gil, Ulsan, South Korea; 13 Institute for Methods and Instrumentation in Synchrotron Radiation Research G-ISRR, Helmholtz-Zentrum Berlin für Materialien und Energie, Albert-Einstein-Strasse 15, 12489 Berlin, Germany; 14 GSI Helmholtzzentrum für Schwerionenforschung, Planckstraße 1, 64291 Darmstadt, Germany; 15 Helmholtz-Institut Jena, Fröbelstieg 3, 07743 Jena, Germany

## Abstract

**Abstract:**

A key requirement for the correct interpretation of high-resolution X-ray spectra is that transition energies are known with high accuracy and precision. We investigate the K-shell features of $\mathrm {Ne}$, $\mathrm {CO}_2$, and $\mathrm {SF}_6$ gases, by measuring their photo ion-yield spectra at the BESSY II synchrotron facility simultaneously with the 1s–*n*p fluorescence emission of He-like ions produced in the Polar-X EBIT. Accurate *ab initio* calculations of transitions in these ions provide the basis of the calibration. While the $\mathrm {CO}_2$ result agrees well with previous measurements, the $\mathrm {SF}_6$ spectrum appears shifted by $\sim$0.5 eV, about twice the uncertainty of the earlier results. Our result for $\mathrm {Ne}$ shows a large departure from earlier results, but may suffer from larger systematic effects than our other measurements. The molecular spectra agree well with our results of time-dependent density functional theory. We find that the statistical uncertainty allows calibrations in the desired range of 1–10 meV, however, systematic contributions still limit the uncertainty to ${\sim }$40–100 meV, mainly due to the temporal stability of the monochromator energy scale. Combining our absolute calibration technique with a relative energy calibration technique such as photoelectron energy spectroscopy will be necessary to realize its full potential of achieving uncertainties as low as 1–10 meV.

**Graphical abstract:**

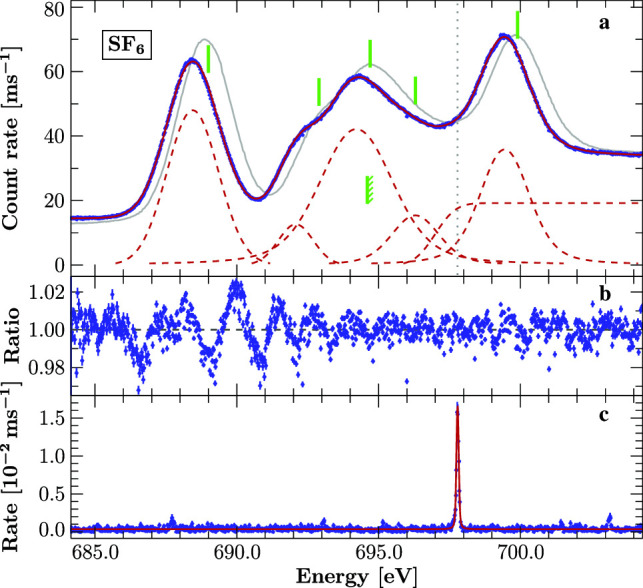

## Introduction

High-resolution astrophysical X-ray spectroscopy has become routine in the last 20 years, with diffraction grating spectrometers on *Chandra* and *XMM-Newton* providing resolving powers of $\Delta \lambda /\lambda \sim 1000$ [1–4]. These instruments have enabled the measurements of the conditions in the emitting plasmas, e.g., through observations of the triplets from He-like ions, precision Doppler velocity and line shape measurements in a variety of astrophysical plasmas, including stellar coronae and winds, cataclysmic variables, X-ray binaries containing neutron stars and black holes, supernova remnants, or outflows in active galactic nuclei [5, 6, 7, 8, 9, 10, 11, e.g.,]. Due to the success of these measurements, future astrophysical X-ray observatories such as *XRISM*, *Athena*, *Arcus*, or *Lynx*, envision spectral resolving powers as high as 5000, implying the ability to accurately determine centroids to 10 ppm, or $3\,\mathrm {km}\,\mathrm {s}^{-1}$ absolute Doppler velocity [12–17]. These instruments will open up the field of spatially resolved, high-resolution X-ray spectroscopy, and will allow scientists to access techniques that are currently not available to X-ray astronomy such as X-ray Fine Structure Absorption measurements for solids [18], the imaging of velocity fields in galaxy clusters [19], or diagnosing the properties of the Warm and Hot Intergalactic Medium [20].

The ground and on-orbit calibration of existing and future instruments as well as the interpretation of the existing and future observations require accurately calibrated atomic transition energies [4, 21, e.g.,]. In one- and two-electron ions, these energies are calculable with part per million (ppm) accuracy for the astrophysically relevant atomic numbers less than 30 [22, 23, 24, 25, e.g.,], and theory has been experimentally benchmarked with precision as good as 10 ppm [26, 27, e.g.,].

Inner shell transition energies in less-ionized species, neutral atoms, molecules, and solids, are far more challenging to calculate accurately, and thus must be obtained experimentally. These experiments, however, rely on existing soft X-ray calibration standards, which have limitations to their accuracy. We recently found a discrepancy in the extensively used standard of the Rydberg transitions of molecular oxygen of almost 0.5 eV [28], thus resolving a tension between astrophysical and laboratory measurements of transitions of atomic oxygen [29], which had been calibrated against this molecular standard [30]. Such discrepancies raise the question of whether other commonly-used soft X-ray standards may have errors of comparable magnitude, given that many such standards are based on similar experimental techniques using electron energy loss spectroscopy (EELS).

Even if the error in the earlier molecular oxygen standard is an outlier, the typical experimental precision of soft X-ray standards obtained with EELS is still of order 0.1 eV (or 100 ppm at 1 keV), which is far too large to fully exploit the capabilities of current and future X-ray astronomical and ground based facilities, and not precise enough for the calibration needs of many future instruments. Modern synchrotron facilities are capable of sufficient photon fluxes and resolving powers that determining centroids of peaks with statistical precision of 1–10 ppm is routine in a variety of experimental disciplines [31, 32, e.g.,], so to the extent that scientific results depend on the absolute transition energies, calibration will often be the limiting factor.

The anticipated high precision of line energy measurements enabled by high spectral resolution coupled with large photon fluxes in future space-based observatories, as well as in high-performance synchrotron beamlines, implies a need to reevaluate soft X-ray transition energies of common elements and materials that have been used for energy calibration using the same accurate standards used by [28]: highly charged ions (HCI) with one or two electrons. To further illustrate the capabilities of these methods, in this paper we present measurements of photoion yield spectra for $\mathrm {CO}_2$ around the oxygen K-edge, $\mathrm {SF}_6$ around the F K-edge, and Ne around its K-edge. These are calibrated using K-shell transitions of He-like N, O, and F, respectively. The remainder of this paper is structured as follows: In Sect. 2 we describe our experimental setup, which combines a synchrotron beamline with an electron beam ion trap (EBIT) to generate the calibrating ions and a gas cell, and discuss the energy calibration and systematic limitations from this setup. In Sect. 3 we present the results of our calibration of the photoionization spectra for neon, $\mathrm {SF}_6$, and $\mathrm {CO}_2$. In order to understand the structure of the molecular edges in greater detail, in Sect. 4 we then compare the experimental results for the molecules with theoretical simulations. We summarize the paper in Sect. 5.

## Experimental setup and data analysis

### Experimental setup

**Fig. 1:**
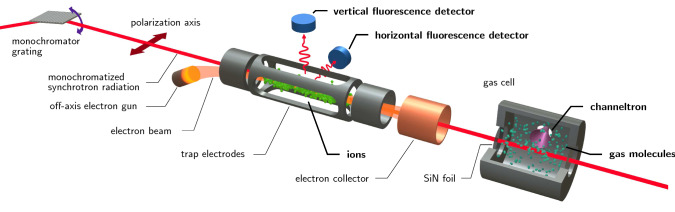
Our scheme for simultaneous measurement of neutral gas photoionization and HCI fluorescence [28, adapted from]. Monochromatic linearly polarized X-rays produced by the synchrotron beamline U49-2/PGM-1 enter the PolarX-EBIT endstation from the left, and excite the HCI. Subsequently, the fluorescence is detected by two silicon drift detectors. The off-axis electron gun allows the synchrotron X-ray beam to pass through to our second endstation, a low-pressure gas cell using a channeltron for detection of photoions

Our experimental setup is depicted in Fig. 1. Monochromatic X-rays from a synchrotron beamline pass through an EBIT, where they interact with HCI. Fluorescence emission from these ions provides the basis for the absolute calibration of the monochromator energy scale in our experiment. The synchrotron radiation passes through the low density plasma in the EBIT with virtually no attenuation, and then enters a gas photoionization cell containing the atoms or molecules under investigation. A channeltron inside the gas cell detects the ion yield due to the interaction of the X-ray beam with the gas. The gas cell with injected gases was operated with a pressure of few $10^-7$ Torr.

Our setup used the PolarX-EBIT [33], which features an off-axis electron gun, enabling the photon beam to pass through the EBIT. The electron beam is tuned to an energy sufficent to ionize atoms entering the trap up to the He-like charge state, but staying below the threshold for K-shell excitations, and also avoiding dielectronic-recombination resonances. The X-ray photons interacting with the ions thus produce a K-shell fluorescence signal that is uncontaminated by X-rays following collisional excitation. We measured this fluorescence signal with silicon-drift detectors (SDDs) that are mounted perpendicular to the electron beam axis.

For H-like and He-like systems it is possible to calculate the transition energies with uncertainties of $\lesssim$1 meV [25]. This *ab initio* provides the absolute calibration reference for our measurements. Since our experiment allows us to measure the fluorescence in the EBIT simultaneously with the ion yield in the gas cell, we avoid problems that are intrinsic to non-simultaneous energy calibrations.

For $\mathrm {Ne}$, $\mathrm {CO}_2$ and $\mathrm {SF}_6$ investigation, we measure the fluorescence of He-like fluorine, nitrogen, and oxygen, respectively. We induce it with soft X-ray photons provided by the BESSY II plane-grating monochromator (PGM) beamline U49-2/PGM-1 [34]. Because of the linear polarization of the beam and the dipolar character of the resonances, there is a strong dependency of the fluorescence on the viewing angle [35]. Therefore, we used two SDDs, one aligned parallel to and the other perpendicular to the polarization axis. Polarization also slightly affects the ion-yield measured in the gas cell. The channeltron was aligned parallel to the polarization axis, but because it was close to the photon beam, it has a finite angular acceptance. The acquired photoion spectra showed features excited by both polarization axes, albeit with a stronger contribution from the parallel axis.

Individual scans for each of the three gases were performed in equidistant energy steps from low to high energies, scanning the photon energy in ranges of 866–871 eV for $\mathrm {Ne}$, 533–540 eV for $\mathrm {CO}_2$, and 684–705 eV for $\mathrm {SF}_6$. At each scan step, the integrated ion-production rate and HCI-fluorescence rates were recorded together with the nominal energy of the beam line. To achieve the highest possible accuracy and minimize uncertainty, the calibration line must lie within the scan range. This was possible for the $\mathrm {CO}_2$ and $\mathrm {SF}_6$ scans. For the $\mathrm {Ne}$ scan, the chosen calibration line was 10 eV lower in energy and had to be recorded in a separate scan. The details of data recording and processing are described in [28], where the same setup was used.

### Energy calibration

The nominal calibration of the beamline wavelength scale uses the grating equation

1 $$\begin{aligned} m N \lambda = \cos \alpha - \cos \beta \end{aligned}$$

where *N* is the line density of the grating and *m* the diffraction order. In our experiment, $m=1$ and $N = 602.4\,\mathrm {mm}^{-1}$. The incident and reflection angles $\alpha$ and $\beta$ are measured with respect to the plane of reflection. These angles are determined from the rotation angles of the mirror and grating using high-precision rotation encoders. Typically, the true wavelength of the beamline has a slight offset from the nominal value derived using the encoder positions. This offset can be corrected using the calibration lines. In many experiments it is common practice to apply a linear offset to wavelength or energy based on a calibration feature. However, since the grating equation is nonlinear, this introduces a systematic error that increases with separation from the calibration feature. Specifically, in energy space the grating equation is given by

2 $$\begin{aligned} E = \frac{h c m N}{\cos \alpha - \cos \beta } \end{aligned}$$

where *h* is Planck’s constant and *c* is the speed of light. We used the defined CODATA 2018 value $hc = 1\,239.841\,984\,\mathrm {eV}\,\mathrm {nm}$ [36, 37]^1^.

The angles comprise two parameters while the selection of energy fixes only one degree of freedom. The remaining degree of freedom is fixed by $\alpha$ and $\beta$ adhering to the fixed-focus condition [38, equation 2, converted to our angle convention]

3 $$\begin{aligned} \sin \beta = c_{\text {ff}} \sin \alpha , \end{aligned}$$

with $c_{\text {ff}}$ set to 2.25 for U49-2/PGM-1. This fixed focus condition ensures that the image of a source at a fixed distance to the grating is projected to a fixed point behind the grating with a scaling, $c_{\text {ff}}$, that is independent of the energy.

Throughout our campaign we found a discrepancy of more than 3 eV at the energy of the $\mathrm {O}^{6+}\,{\mathrm{1s}}^2\,{}^1S_0 \rightarrow 1s2p\,{}^1P_1$ transition. We will call this line O$_{\text {w}}$ in the following [39]; its theoretical energy is 573.961 eV [23]. Assuming that the accuracy of the angle steps is stable (at least over single scans, containing 100–1000 steps each), this discrepancy must be due to an offset in the angles such that $\alpha = \alpha ' + \Delta \alpha$, $\beta = \beta ' + \Delta \beta$, where $\alpha '$ and $\beta '$ are the incident and reflected angles of the photons as reported by the beamline.

A single calibration feature only permits to determine either $\Delta \alpha$ or $\Delta \beta$. A natural choice for their relation is to have the corrected values fulfill the fixed-focus condition, which can be approximately achieved through

4 $$\begin{aligned} \Delta \alpha c_{\text {ff}} = \frac{\cos \beta '}{\cos \alpha '}\Delta \beta . \end{aligned}$$

Although we emphasize that this choice is not *a priori* theoretically motivated, we found that the derived energy scale is not sensitive to a particular relation between $\Delta \alpha$ and $\Delta \beta$ if both are sufficiently small, and the calibration is applied to an appropriately small energy range. The reason is that small changes of $\alpha$ or $\beta$ have the same effect, that is, shifting the energy scale. It is only for large changes of $\alpha$ or $\beta$ that the slope of the calibration changes.

The final calibration of the energy of the gas cell measurements is achieved through a simultaneous fit to both the photoion yield spectrum in the gas cell and the fluorescence spectrum in the EBIT. Using theoretical values for the energies of the fluorescence lines, the free parameters of the fit are the angular shifts of $\alpha$ and $\beta$, the energies of the photoionization resonances in the gas cell spectrum, and their respective widths. Each fluorescence line and photoionization resonance is modeled with a Voigt profile, with the Gaussian $\sigma$ and Lorentzian $\Gamma$ parameters representing a combination of instrument profile, natural linewidth, and thermal Doppler broadening; both spectra also include background which we model with a energy independent constant. In the fluorescence spectrum, it is mainly caused by the high-energy tail of the pulse height distribution of low-energy photons detected by the SDDs. In the photoionization spectra, it results from residual gases that do not have resonant features in the bands of interest, and can thus be treated as a constant contribution to the detected signal.

**Fig. 2:**
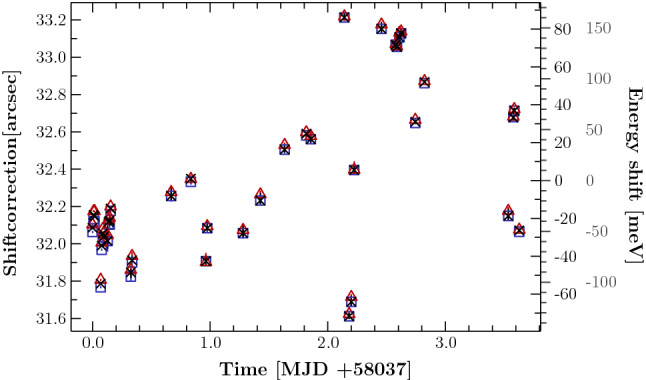
Measured position of the $\hbox {O}_{\mathrm{w}}$ resonance in terms of the angle correction at different times during the campaign. Black crosses and red triangles respectively indicate the reported energy after and before the photon measurement, and blue squares their mean (see text for details). The right scale shows the translated energy spread around the mean of all measurements at the O$_{\text {w}}$ line (inner) and the Ne 1s–3p transition (outer). Measurements of this line were mainly used to benchmark the X-ray beam at different settings causing a variation in the uncertainty

All of the fits are evaluated using the Cash statistic [40], a version of the likelihood ratio test that is appropriate for Poisson distributed data. Since we model the calibration and ionization data simultaneously, it is possible to estimate confidence intervals for our parameters of interest by confidence search [41]. These intervals describe the total statistical uncertainty for each emission line in the photoionization spectrum, including the one from the calibration measurement (but excluding systematic uncertainties, as discussed below). The confidence intervals derived using this approach cover the 90% uncertainty interval and are typically in the range between 1 to 10 meV.

### Systematic limitations

The calibration uncertainty of the photoionization spectra is dominated by systematic terms. As discussed in Sects. 2 and 2.2, the statistical uncertainties on the calibration are typically smaller than 10 meV, while the theoretical uncertainties in our calibration line energies are smaller than 1 meV.

In our current setup, a contribution to the uncertainty much larger than those comes from the stability of the beamline. We can estimate the long-term variability of the beamline from scans measuring the same transition throughout the measurement campaign. The fluctuation of the shift parameter, $\Delta \beta$, is shown in Fig. 2 for repeated measurements of the $\hbox {O}_{\mathrm{w}}$. On the right-hand *y*-axis, we display the corresponding effect of such an angular shift on the energy calibration at the $\hbox {O}_{\mathrm{w}}$ line (574 eV) and the neon K-edge (870 eV). In our experiment we requested the reported monochromator energy and angle settings twice for each scan step; once before data acquisition with the SDD and once after. We found that the reported energy values before the SDD acquisition often showed unreasonably high fluctuations, probably attributable to the relaxation of the beamline to the selected energy immediately after moving the monochromator, even after the allowed settling time. For this reason we only used the values reported after each scan step for our further analysis. As more extensively discussed in the supplemental material of [28], based on repeated scans of multiple closely-spaced photoionization lines in the gas cell, and also on studies of the shapes of single fluorescence lines in the SDD, we conclude that such large shifts do not occur in single scans; however, shifts of up to 40 meV can be expected near $\hbox {O}_{\mathrm{w}}$. Given that the energy shifts for a fixed angular shift become larger at higher energies, we estimate that the systematic energy shift at the Ne K-edge can be as high as 100 meV; we discuss this further in Sect. 3.1.

## Energy calibration of $\mathrm {Ne}$, $\mathrm {CO}_2$, and $\mathrm {SF}_6$

We now discuss the results of modeling each of the photoionization spectra measured for neon, $\mathrm {SF}_6$ and $\mathrm {CO}_2$.

### $\mathrm {Ne}$ Rydberg series

We calibrated our scan of atomic neon using the $\hbox {F}^{7+}\,K_\beta$ transition ($E_{\text {F}\,K_\beta }$ = 857.5108(7) eV, [25]).

This line was scanned before and after the actual ionization measurement of neon and, therefore, not simultaneously. As discussed above, this adds an additional uncertainty which can, in principle, be as large as 150 meV (Fig. 2). The angular shift corrections measured for the two $\hbox {F}^{7+}\,K_\beta$ scans differ by $0.2''$, corresponding to about 30 meV at $E_{\text {F}\,K_\beta }$. Instead of using the averaged shift correction as obtained from both calibrations, we weigh the shift correction of the neon data with Student’s *t* distribution [42]. In this way we can estimate the statistical uncertainty due to the variation of the calibration by assuming that these are drawn from a normal distribution. Just accounting for statistical variations, the resulting 90% confidence interval for the energy of the neon lines is $\pm 15$ meV. The systematic uncertainty can not be quantified directly but can be deduced by comparison to previous experiments. Overall we estimate a 100 meV calibration uncertainty.

**Fig. 3:**
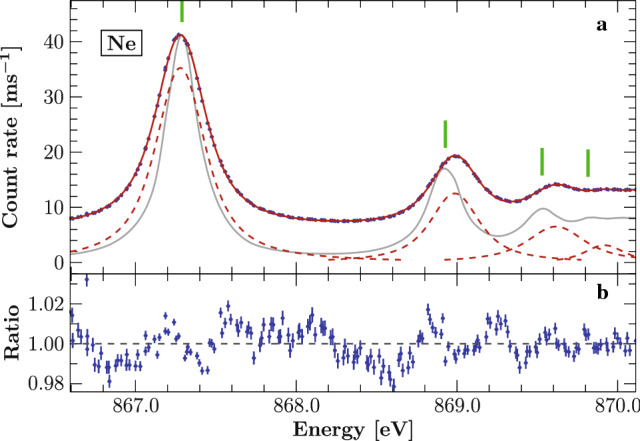
**a:** Neon spectrum (blue points) calibrated using measurements of F $K_\beta$ in scans before and after the photoionization measurements. The neon emission lines are modeled by a sequence of Voigt profiles to determine the peak positions (red solid line, components red dashed lines). The green vertical bars indicate the line positions as reported in [43] and the gray solid line outlines their data (scaled to the 1s–3p transition). **b:** The ratio between the data and the model. The calibration line was modeled with Voigt parameters $\sigma = 0.172$ eV and $\Gamma < 0.001$ eV

The Rydberg series (Fig. 3) is modeled by a set of five Voigt profiles without constraints on the line shape parameters. The scan range does not reach up to the series limit such that it is not possible to include a component for the edge without constraints on its location. The model used by [31], where the positions are constrained by a Rydberg series modified by a quantum defect, is not describing our data to a satisfying level. Therefore we did not constrain the line positions and we also did not include a component for the ionization edge. Ignoring contributions of the ionization edge to the high energy part of the scan causes the fifth Voigt profile to model all contributions from higher Rydberg transitions and the ionization edge. This behavior of the model can have an effect on the position of the 1s-6p line, but we expect that the effect on the lower transitions is only marginal and below the uncertainty. The resulting model is shown in Fig. 3 and has only a few residual patterns left. A part of these residuals can be attributed to the uncertainty (or jitter) of the reconstructed energy grid. We verify this by fitting the same model to the data using the nominal energy grid. Here the residuals cluster around the wings of the model lines since a jitter in the energy grid has a larger impact at energies the derivative of the model has a larger absolute value. For the reconstructed energy grid these residual patterns are stretched over a wider energy range.

Our determined line positions are given in Table 1. We compare these results with those found in [43], which have been calibrated using the measured 1s–3p transition of [44]. The agreement of the first line is very good, while the subsequent lines diverge more with higher energy. The difference of order 50-100 meV in the higher-*n* lines is consistent with the amplitude of drift observed in the energy scale of U49-2/PGM-1, as discussed in the supplemental material of [28].

**Table 1:** Measured $\mathrm {Ne}$ Rydberg transitions lines calibrated against the F $K_\beta$ line

Transition	Energy (eV)	
	This $\hbox {work}^{\mathrm{(a)}}$	Müller et al. [43]$^{\mathrm{(b)}}$
1s–3p	867.278	867.290
1s–4p	868.980	868.928
1s–5p	869.620	869.530
1s–6p	869.920	869.815

$^{\mathrm{(a)}}$ Calibrated against F $K_\beta$ (857.5108(7) eV, [25])

$^{\mathrm{(b)}}$ Calibrated against neon 1s–3p [44]

Statistical uncertainties of the peak positions are $\pm 15$ meV but are largely exceeded by systematic variations of up to 100 meV (see text). Recent high resolution measurements of the neon Rydberg series are given for comparison [43]

### $\mathrm {CO}_2$ Oxygen K-edge

We measured the photoionization yield of $\mathrm {CO}_2$ in the range 533 to 540 eV. The calibration of the energy grid is based on the theoretical predictions of the He-like nitrogen transition N $K_\epsilon$ [25, $E_{\text {N}\,K_\epsilon } = 538.4924(3)$ eV,].

**Fig. 4:**
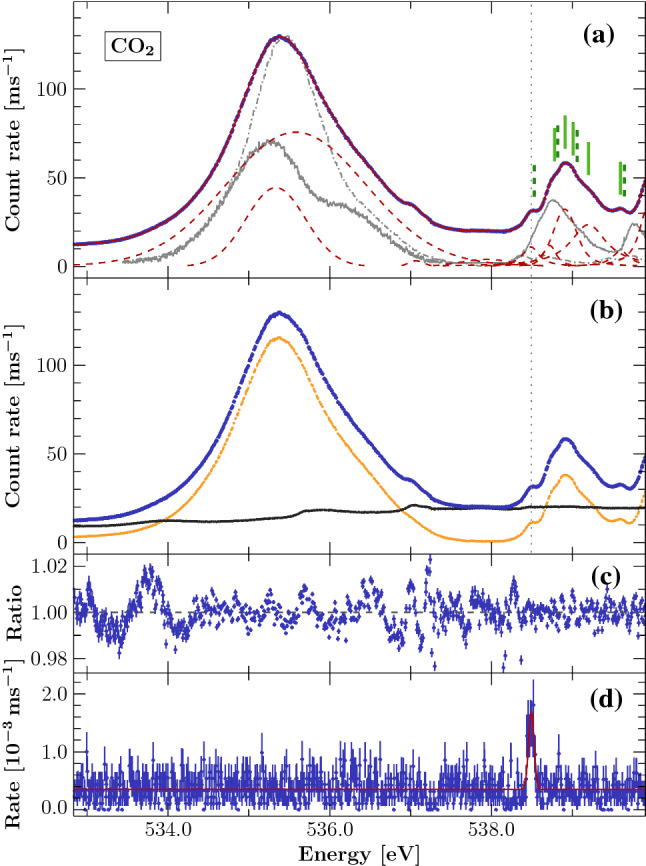
**a:** Calibrated $\mathrm {CO}_2$ spectrum (blue points) based on simultaneous measurements of N $K_\epsilon$. Emission lines are modeled by Voigt profiles (red solid line, components red dashed lines). Model components may represent multiple unresolved transitions. The green lines indicate the transition energies in the Rydberg complex reported in [45] for the two resolved symmetry directions 0$^\circ$ (solid) and 90$^\circ$ (dashed). Solid gray and dashed-dotted gray line indicated their data, for 0$^\circ$ and 90$^\circ$, respectively. The dotted gray vertical line indicates the location of the calibration line for our data. **b:** Residual water vapor in the gas cell adds additional spectral features. The background was estimated from data from a second gas cell (black points); The corrected spectrum (orange points) shows the difference between the data of the two gas cells. The uncorrected data is again given here for reference (blue points). **c:** Residuals between model and data as ratio. **d:** Sum of the fluorescence spectra produced in the EBIT measured by the two SDDs. The calibration line was modeled with Voigt parameters $\sigma = 0.082$ eV and $\Gamma < 0.001$ eV

This line was measured simultaneously with the ionization spectrum, thus significantly reducing the overall uncertainty. The measured $\mathrm {CO}_2$ spectrum is rich, featuring (in our spectrum) unresolved vibrational structure, and showing a mixture of lines from both polarizations [45, 46]. To determine the transition energies, we empirically modeled the spectrum with a set of 10 Voigt profiles, which in many cases represent blends of unresolved emission lines. The choice of 10 lines is only supported by the number of features which are identifiable by eye. The background is modeled with an energy independent constant. Figure 4 shows the calibrated data and best fit model. This best fit model is reasonably good, with residuals comparable to the neon measurement. On closer inspection, the spectrum shows a rich structure which is only barely resolved in our data but clearly visible in recent resonant inelastic X-ray scattering (RIXS) measurements [47].

This large number of parameters in the empirical model poses a difficult problem for classical fit algorithms, especially with the addition of the calibration function itself. To find the minimum of this function, we made use of the Markov Chain Monte Carlo (MCMC) algorithm proposed in [48]. It explores the probabilistic parameter space, and additionally gives the parameter uncertainty. The resulting 90% confidence for the first 9 line profiles is $\pm 3$ meV. The tenth line is only partly covered by the scan, and therefore not well constrained.

We list the resulting line positions in Table 2, where the assignments are by strongest contribution to our model based on the measurement of [46]. The resonance peak shows two main features [45, e.g.,] generally associated to the valence orbital and contribution from the 3s$\sigma$ state. A small emission line is visible at the shoulder of the resonance peak together with an excess of events between the resonance and the Rydberg complex compared to recent high resolution RIXS measurements [47]. This excess can be attributed to residual water vapor in the gas cell.

**Table 2:** $\mathrm {CO}_2$ measured transitions in our calibration

Transition	Energy (eV)		
	This work$^{{\mathrm{(a)}}}$	Okada [46]$^{{\mathrm{(b)}}}$	Adachi [45]
$$\left. \begin{array}{l} \pi ^*\\ \hbox {3s}\sigma \end{array}\right\} $$	$$\begin{array}{l} 535.334\\ 535.582\end{array}$$	535.4$^{\mathrm{(c)}}$	535.4$^{\mathrm{(c)}}$
contam.	537.069	–	–
contam.	537.937	–	–
3p$\pi _u$	538.487	538.53	538.53
$$\left. \begin{array}{l} \hbox {3p}\sigma _u\\ \hbox {3p}\pi _u\end{array}\right\} $$	538.720	$$\left\{ \begin{array}{l} 538.78\\ 538.83\end{array}\right. $$	$$\left\{ \begin{array}{l} 538.78\\ 538.82\end{array}\right. $$
$$\left. \begin{array}{l} \hbox {4s}\sigma _g\\ \hbox {3p}\sigma _u\end{array}\right\} $$	538.908	$$\left\{ \begin{array}{l}538.93\\ 539.04\end{array}\right. $$	$$\left\{ \begin{array}{l}538.91\\ 539.06\end{array}\right. $$
$$\left. \begin{array}{l} \hbox {4s}\sigma _g\\ \hbox {3p}\sigma _u\end{array}\right\} $$	539.197	$$\left\{ \begin{array}{l}539.18\\ 539.30\end{array}\right. $$	539.20
3d$\pi _g$	539.595	539.67	539.64

$^{{\mathrm{(a)}}}$ Calibrated against N $K_\epsilon$ (538.4924(3) eV, [25])

$^{{\mathrm{(b)}}}$ calibrated against $\mathrm {CO}_2$ transitions from [49]

$^{\mathrm{(c)}}$ Unresolved blend of $\pi ^*$ and 3s$\sigma$, reported in [50]

For comparison the experimental values of [45, 46] are listed. Assignments are based on the assignments of [46]. Line blending and mixing is indicated by braces. We estimate the uncertainty of our energy scale to 40 meV (see text)

An estimate of the residual gas is obtained from a second gas cell operated upstream of the first cell and separated from it by a thin SiN window. The second cell was operated with no sample gas injection, and therefore all photoions detected are from background gases. In principle, the background gas composition in the two cells may be different. However, due to insufficient bakeout, the residual gas in our vacuum chambers was dominated by water vapor, as can be seen by comparing the features in the background gas spectrum to previously published measurements of water vapor [51]. As indicated in Fig. 4, we see that the residual gas spectrum explains the feature on the high energy side of the $\pi ^*$ resonance as well as the unexpectedly high amplitude of the continuum between the resonance and the Rydberg complex. Because we could not be certain that the amplitude of the background spectrum was the same in both cells, we cannot use the second cell to correct the first. However, we can try modeling the background in the first cell based on the spectrum of the second and assess the impact on our fit results. We found that the energy of the $3s\sigma$ peak moved to slightly higher energy, while the other peak energies were unaffected. We attribute the remaining residuals in the $\pi ^*$ resonance to a combination of unresolved vibrational structure [51, 52] and a possible non-ideal instrument lineshape. The dominant uncertainty in the transition energy determination is drift in the monochromator energy scale. Based on the analysis in [28] we estimate this uncertainty to be 40 meV for these lines.

Direct comparison of the results with the literature is in general not possible due to the blending of transitions. However, the 3p$\pi _u$ transition is easily identifiable in our scan as well as in recent measurements [45–47]. Additionally, its energy is very close to our calibration line and, hence it has much smaller shifts due to drift. Using this as a reliable reference energy, we see that the Rydberg complex from [46] appears at slightly higher energies. The result of [46], calibrated using $\mathrm {CO}_2$ measurements from [49], which in turn are calibrated against 0$_2$ measuremnts from [53]^2^. Given the uncertainties of 100-200 meV in their work, we conclude that our measurements of the transition energy of 3p$\pi _u$ agree. Similar EELS measurements [55] also place the $\pi ^*$ resonance at a higher energy, but comparison of the Rydberg complex is difficult due to their limited energy resolution and lack of polarization selectivity.

### $\mathrm {SF}_6$ fluorine K-edge

We scanned the fluorine K-edge of $\mathrm {SF}_6$ in the range from 685 to 705 eV measuring the photoionization yield of the gas in the gas cell. The calibration is based on the O $K_\gamma$ transition of He-like oxygen ($E_{\text {O K}_\gamma } = 697.7859(5)$ eV, [25]).

**Fig. 5:**
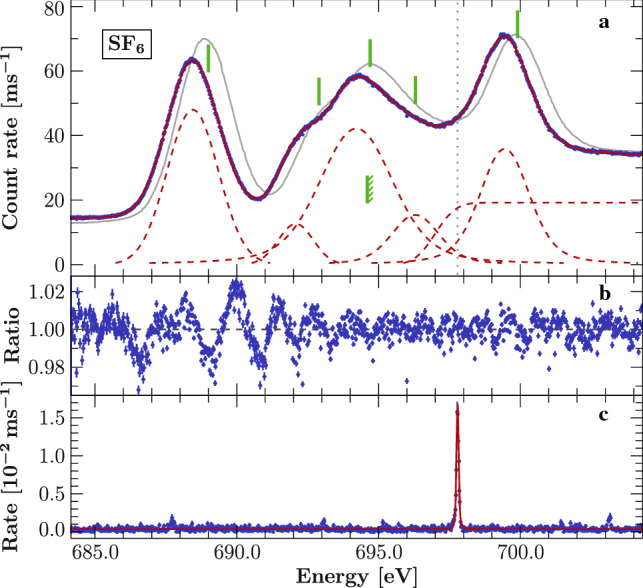
**a:** $\mathrm {SF}_6$ photionization spectrum (blue points) calibrated by simultaneous measurement of the O $K_\gamma$ transition and modeled by five Voigt profiles and one error function (red solid line, components red dashed lines). The position of the calibration line is indicated by the dotted vertical line. The solid green lines indicate the measured peak positions from [56] and the edge as measured in [57] (indicated with diagonal marks). The gray curve outlines the measurements of [56] scaled to match the present results. **b:** Ratio between the data and the best-fit model. **c:** Sum of fluorescence spectra measured with the two SDDs. The calibration line is modeled with the Voigt parameters $\sigma = 0.079$ eV and $\Gamma = 0.031$ eV

Following [56], we describe the spectrum by a sequence of five Voigt profiles together with an error function to account for the photoelectric absorption edge at the Rydberg series limit. In [56] an arctangent function convolved with a Gaussian was used to describe the edge, but given our resolution we cannot discriminate between these choices. Hence, for simplicity we used only an error function to model the edge. The calibrated data and resulting best fit are displayed in Fig. 5. Similarly to [56], we have to add a line around 696 eV, otherwise an excess of events remains visible in the data in comparison with the model. Further justification for emission at this energy is given by theoretical predictions (see Sect. 4). From the confidence calculations, we estimate the 90% uncertainty to $\pm 3$ meV for the line positions; the edge energy has an uncertainty of $+2$ meV. The systematic uncertainty is dominated by drift in the beamline energy scale, and based on [28], we estimate it to be 60 meV.

Overall, the empirical model describes the data very well. However, the lowest energy line has significant residuals. These residuals may originate from a combination of unresolved vibrational structure [51, 52] and a possibly non-ideal instrument lineshape.

The spectrum has been measured several times in the past with varying results [56, 57, 58–60, 61, e.g.,]. Many of the EELS measurements use the measurements in [60] for calibration, which itself is based on measurements from [62]. The EELS measurements have a difference of ${\sim }500\,\mathrm {meV}$ with our result, two to three times more than their claimed uncertainty, but in agreement with the discrepancy in [28]. The photoionization measurements from [56] also show a shift to higher energies, but their calibration was provided only by the used beamline. The results of this work together with selected previous results are given in Table 3. It is evident that the often used value of the edge energy as reported in [57] is not compatible with our results (indicated in Fig. 5). A similar observation can be made from the spectrum given in [56]; however, their edge location is not quantified.

**Table 3:** Calibrated $\mathrm {SF}_6$ transitions and comparison to other publications

This work			Energy (eV)			
Symmetry	Experiment$^{{\mathrm{(a)}}}$	Theory	Other experiments			
			Eustatiu$^{{\mathrm{(b)}}}$	Francis$^{{\mathrm{(c)}}}$	Hudson$^{{\mathrm{(d)}}}$	Hitchcock$^{{\mathrm{(e)}}}$
${\hbox {a}_{\mathrm{1g}}}$	688.448	–	687.9	688.0	689.0	688.0
$$\left. \begin{array}{l} \underline{\hbox {a}_{\mathrm{1g}}}\\ \hbox {t}_{\mathrm{1u}}\end{array}\right\} $$	692.082	$$\left\{ \begin{array}{l} \underline{691.59}\\ 692.23\end{array}\right. $$	691.4	692.4	692.9	692.6
$$\left. \begin{array}{l} \hbox {t}_{\mathrm{1u}}\\ \hbox {e}_{\mathrm{g}}\\ \hbox {t}_{\mathrm{2g}}\\ \underline{\hbox {t}_{\mathrm{1u}}}\end{array}\right\} $$	694.217	$$\left\{ \begin{array}{l} 693.67\\ 693.81\\ 693.95\\ \underline{694.19}\end{array}\right. $$	693.5	694.0	694.7	694.6
$$\left. \begin{array}{l} \hbox {t}_{\mathrm{1u}}\\ \hbox {t}_{\mathrm{1u}}\\ \underline{\hbox {t}_{\mathrm{2u}}}\end{array}\right\} $$	696.296	$$\left\{ \begin{array}{l} 695.11\\ 695.43\\ \underline{695.69}\end{array}\right. $$	–	–	696.3	–
I.P.	696.998	–	694.6$^{{\mathrm{(f)}}}$	694.6$^{{\mathrm{(f)}}}$	–	694.6$^{{\mathrm{(f)}}}$
${\hbox {t}_{\mathrm{2g}}}$	699.446	699.51	698.8	698.9	699.9	699.1

$^{{\mathrm{(a)}}}$ Calibrated against O $K_\gamma$ [25, 697.7859(5) eV,].    $^{{\mathrm{(b)}}}$ Eustatius et al. [58]

$^{\mathrm{(c)}}$ Francis et al. [59].   $^{\mathrm{(d)}}$ Hudson et al. [56]

$^{\mathrm{(e)}}$ Hitchcock & Brion [60].    $^{\mathrm{(f)}}$ Determined from XPS [57]

Theory values obtained by TDDFT calculations (see Sect. 4. The first excitation ($\hbox {a}_{\text {1g}}$) is chosen to align with the experiment. Assignments are based on these calculations, where the largest contribution to each spectral feature is underlined. Line blends are indicated by braces. For comparison, the results of selected publications are listed. We estimate the uncertainty of our energy scale to 60 meV

## Modeling K-edge absorption spectra from first principles

**Fig. 6:**
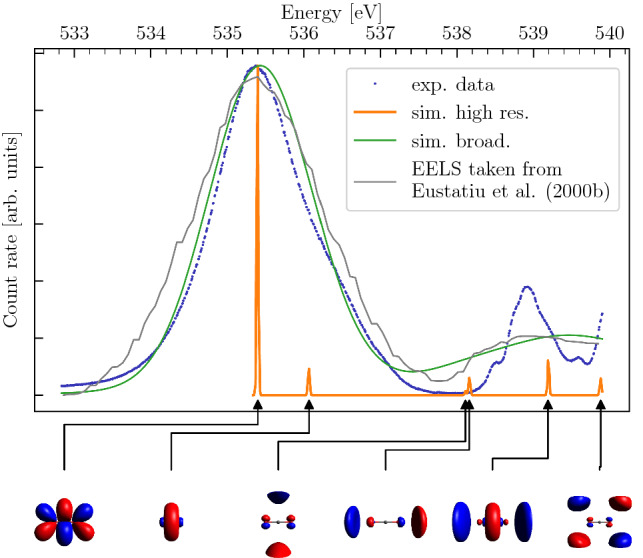
$\mathrm {CO}_2$ X-ray absorption spectrum from TDDFT calculations for oxygen K-edge. Highly resolved peaks were numerically broadened to visualize agreement of simulation and experiment. Experimental data are shown after subtraction of contamination. Additional data extracted from [55] with aligned first peak are depicted for comparison. The baseline of all of these spectra has been unified. Corresponding molecular orbitals are plotted below

**Fig. 7:**
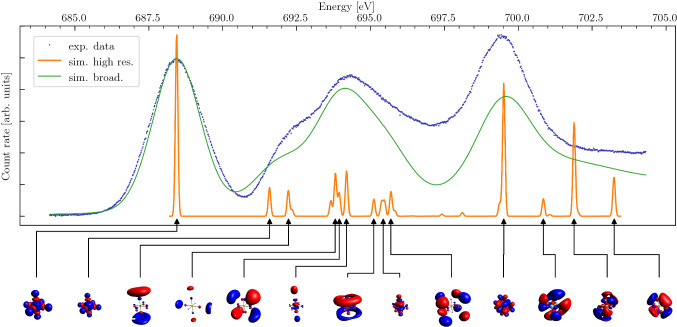
$\hbox {SF}_{6}$ X-ray absorption spectrum from TDDFT calculations for fluorine K-edge. Highly resolved peaks are numerically broadened to visualize agreement of simulation and experiment. Experimental data are shifted to the baseline. The corresponding molecular orbitals are plotted below the *x*-axis

In order to assist in the interpretation of the experimental data we performed *ab initio* TDDFT simulations of the oxygen K-edge excitations in $\mathrm {CO}_2$ (shown in Fig. 6) and fluorine K-edge in $\mathrm {SF}_6$ (shown in Fig. 7). The computation of the molecular orbitals associated with the excited states allows us to attach symmetry labels to the experimental peaks. Moreover, the arbitrarily high resolution of the simulated spectra can help to understand whether observed peaks originate from single broadened transition lines or if there is a richer spectral structure which cannot be resolved experimentally.

### Calculation details

For the simulation we employ Time-Dependent Density Functional Theory (TDDFT) as implemented in the ORCA quantum chemistry code [63]. We used a minimally augmented diffuse quadruple zeta basis set ma-def2-QZVPP [64, 65] and the Coulomb fitting auxiliary basis def2/J in combination with the hybrid functional PBEh$\alpha$ [66]. All calculations employed the RIJCOSX approximation [67]. Moreover, we considered only purely electronic effects and neglected vibrational modes of the molecules. The only free parameter of the DFT simulation is the mixing factor $\alpha$ of the hybrid functional, for which we found the best agreement with the present experiments at a value of $\alpha =35\,\%$. The infinitely sharp transitions of the TDDFT simulation were subsequently broadened (convolved) for comparison with experimental data. Following common procedure, we employed an energy-independent Gaussian of 1.8 eV (i.e., accounting for measurement effects) and an energy-dependent Lorentzian (i.e., life time broadening) of the order of 0.10–4.47 eV. Since the energy offset of the TDDFT spectra is known to be unreliable, following common practice the simulated spectra were shifted to align the lowest lying excitation with the experimental data.

### General remarks

While our TDDFT calculation includes core-hole effects [68, 69] beyond a simple mean-field limit, excitonic multiplet splittings of the excited states are negligible in K-edges (as opposed to, e.g., transition metal L- or rare-earth M-edges). Therefore, a single-particle picture can be used to interpret the excitations as the promotion of an oxygen or fluorine 1s core electron into “unoccupied” molecular orbitals. We can exploit this single-particle nature and associate to each peak in the spectrum a corresponding single electron molecular-orbital computed from the self-consistent DFT (and plotted with the Avogadro program [70]). Its symmetry (and degeneracy) then allows us to classify the excitations in terms of irreducible representations of the molecules point-group. For more details on approximation and simulation strategies for (especially oxygen) K-edge absorption in atoms, molecules, and solids, we refer the interested reader to a recent review [71].

### The oxygen K-edge of $\mathrm {CO}_2$

In Fig. 6 we show the comparison of simulation (orange and green lines) and experiment together with the earlier EELS data [55]. The first part of the K-edge is dominated by the well known transition into the $\pi _u$ orbital at around 535.4 eV. The peaks at higher energies are typically assigned to Rydberg transitions. In this energy region we get a satisfactory agreement in terms of the overall relative spectral weight at a low resolution (see broadened simulation vs. EELS in Fig. 6). However, the simulation misses the splitting of peaks picked up by higher resolution experiments. An explanation might be our neglect of vibrational modes. Indeed, $\mathrm {CO}_2$ as a linear ($D_{\infty h}$) molecule, is a prime candidate even for irregular vibrational structure due to the Renner-Teller effect [71]. Moreover, we point out that the simulated spectra correspond to absorption with unpolarized light and can thus only be directly compared to the EELS data [55]. We do not account for matrix elements in the transition that account for polarization dependence in the new experimental data.

The summary of our *ab initio* symmetry classification of the peaks can be found in Table 4.

### The fluorine K-edge in $\mathrm {SF}_6$

In Fig. 7 we compare our simulation to the experiment. In contrast to the oxygen K-edge of $\mathrm {CO}_2$, the fluorine K-edge in octahedral ($O_h$) $\mathrm {SF}_6$ is not dominated by a single transition and has a comparable spectral weight in three main structures between 685 eV and 705 eV. With the same calculation parameters that we used for $\mathrm {CO}_2$, we find an overall satisfactory agreement with experiment. The comparison reveals that particularly the central double-peak structure around 693.5 eV might originate from a variety of transitions which are, however, not resolved in experiment. In Table 3 we provide a comprehensive list of energies and symmetry character of the transitions.

**Table 4:** Assignment of irreps for transition orbitals with corresponding transition energy in K-edge excitation of $\mathrm {CO}_2$

irrep	energy [eV]
$e_{1u}$ ($\pi _u$)	535.4
$a_{1g}$ ($\sigma _g^+$)	536.07
$e_{1u}$ ($\pi _u$)	538.11
$a_{2u}$ ($\sigma _u^-$)	538.16
$a_{1g}$ ($\sigma _g^+$)	539.19
$e_{1g}$ ($\pi _g$)	539.88

## Conclusions

We used a newly introduced experimental setup to provide precise calibration references in the soft X-ray regime. A careful statistical analysis shows that the resulting energy calibration can in principle provide an accuracy of 1–10 meV (at energies in the 500–800 eV range). The resulting calibrations have no dependence on previous measurements and therefore do not carry any legacy uncertainty present in other measurements. The achieved accuracy is limited by significant systematic uncertainties that exceed the statistical uncertainties by almost an order of magnitude. We performed several measurements of molecular absorption spectra that are commonly used for energy calibration. The results for $\mathrm {CO}_2$ show relatively good agreement with previous ones; for $\mathrm {SF}_6$, we see a shift similar to that found in [28]. Significant differences appearing in the $\mathrm {Ne}$ measurement compared to earlier works might be partly an artefact of the non-simultaneous measurement of the calibration, and require further investigation. Our theoretical simulations of the $\mathrm {SF}_6$ spectrum, although consisting of numerous features, also show fairly good qualitative agreement with the data. We are able to attribute a much richer structure underlying the measured spectral weight at 691–697 eV, supporting [56] in contrast to other works [58–60]. For $\mathrm {CO}_2$, the experimental spectra exhibit several features not captured by the simulations. We attribute these differences to us neglecting polarization dependence (dichroism) and vibrational effects. Since such vibrational and symmetry-resolving effects do not influence the relative positions of the peaks due to optical excitations, explicitly correlated methods from many-body perturbation theory [72, e.g., Bethe-Salpeter formalism;] may improve predictions from theory.

Despite the systematic effects still present in our current experiment, we have reduced the overall uncertainty in comparison with various previous measurements. For further investigations aiming at reaching a statistically dominated accuracy, it is necessary to follow in time small relative energy shift of the photon beam energy selected by the monochromator. This could be achieved by, e.g., photoemission spectroscopy simultaneously performed with the photoionization measurements, and would remove any dependency caused by the beamline.

The accuracy of theoretical calculations for few-electron HCI surpasses that of any other soft X-ray standards, and thus our method can in principle provide references at the level of $\sim$50 meV for this range. Such references will find various applications in different fields of research, and help, as shown in this work, assessing the accuracy of calculation for molecular systems. Crucially, our calibration method and the present results address essential needs of upcoming X-ray astrophysics missions.

## Data Availability

This manuscript has no associated data or the data will not be deposited. [Authors’ comment:The data that support the findings of this study are available from the corresponding author, J. S., upon reasonable request.]

## Footnotes

1 The nominal energies reported by the beamline use $hc = 1\,239.86\,\mathrm {eV}\,\mathrm {nm}$.

2 For the calibration with respect to $\hbox {O}_{2}$, [49] references [54]. However, the given value is actually obtained from [53].

## Data Availability Statement

This manuscript has no associated data or the data will not be deposited. [Authors’ comment: The data that support the findings of this study are available from the corresponding author, J. S., upon reasonable request.].
